# The Impact of Childhood Adversity on Life Course Alcohol Use Patterns and Health Status Among People Living with HIV

**DOI:** 10.1007/s10461-024-04368-1

**Published:** 2024-06-22

**Authors:** Rayna E. Gasik, Aubrey Spriggs Madkour, Simone J. Skeen, Gretchen Clum, Tishawn Francis, Erica Felker-Kantor, Tekeda Ferguson, David A. Welsh, Patricia E. Molina, Katherine P. Theall

**Affiliations:** 1 School of Public Health and Tropical Medicine, Tulane University, New Orleans, LA USA; 2 Department of Psychology, Hunter College, City University of New York, New York, NY USA; 3 Department of Epidemiology, School of Public Health, Louisiana State University Health Sciences Center, New Orleans, LA USA; 4 Section of Pulmonary/Critical Care, School of Medicine, Louisiana State University Health Sciences Center, New Orleans, LA USA; 5 Department of Physiology, School of Medicine, Louisiana State University Health Sciences Center, New Orleans, LA USA; 6 Comprehensive Alcohol Research Center, School of Medicine, Louisiana State University Health Sciences Center, New Orleans, LA USA; 7 The Blood Center, New Orleans, LA USA

**Keywords:** Adverse childhood experiences, Childhood economic hardship, Mental health, Alcohol use, HIV

## Abstract

Adverse childhood experiences (ACEs) and financial hardship are associated with increased likelihood of heavier alcohol use and health challenges in adulthood among persons living with HIV (PWH). We examined whether retrospectively captured lifetime drinking trajectories are a pathway through which childhood hardships affect current health in a sample of 365 adult PWH. Childhood economic hardship and ACEs were used as main predictors. Measures of alcohol use included age at first drink and lifetime drinking trajectories. Health indicators included health-related quality of life, frailty, number of comorbidities, and symptoms of anxiety, depression, and post-traumatic stress disorder (PTSD). Structural equation modeling (SEM) was applied to estimate both direct and indirect pathways between childhood hardship and physical and mental health. Participants were mostly male; Black (84%); and averaged 48 years of age. SEM results supported both direct and indirect pathways between childhood experiences and adult health. ACEs were connected to physical health directly and mental health both directly and indirectly through age at first drink and drinking heaviness during ages 10–20. Childhood economic hardship related to mental health indirectly through higher drinking levels during ages 10–20. Childhood adverse experiences, economic hardship, and early drinking patterns appear to accumulate, resulting in later life physical and mental health concerns for PWH. Findings support taking a life course approach to health. This includes considering individual trauma histories in HIV care engagement and taking preventative approaches which support the economic and social well-being of vulnerable children to improve health in subsequent decades.

## Introduction

People living with HIV (PWH) are more likely to have experienced traumatic events, including adverse childhood experiences (ACEs), compared to seronegative individuals [1–4]. HIV-seropositive individuals are 1.5–2.0 times more likely to have experienced childhood physical or sexual abuse than seronegative populations [2]. In a study of 611 in-care PWH in the southern United States, over 50% had experienced at least one form of physical abuse or neglect prior to age 13 [5]. Childhood adversity includes both the witnessing or experiencing of traumatic events (ACEs) and childhood economic hardship (CEH), a condition where a child’s family struggles financially to cover basic needs such as food and housing. Several studies have shown that childhood adversity not only contributes to the transmission of HIV but is also associated with the progression of the disease, poor quality of life, poor antiretroviral therapy (ART) adherence, increased healthcare utilization, and higher HIV-related and all-cause mortality rates among PWH [1–3]. Research indicates that early life exposure to adversities such as poverty, neglect, or abuse is linked to negative health outcomes through changes in social information processing, emotional responses, and biological aging (younger initiation of puberty and cellular aging) [6].

Childhood adversity (ACEs and CEH) is linked to poorer physical and mental health outcomes in adulthood among many PWH [2–4, 6–10]. Mental health challenges such as anxiety and depressive symptoms, post-traumatic stress and formally diagnosed PTSD, are linked to early-life trauma [2, 11]. Physical health outcomes associated with ACEs, trauma, and CEH are documented as well. These include elevated rates of sexually transmitted infection (STI) acquisition, obesity, cardiovascular disease, and diabetes [12–14]. Experiencing a greater number of ACEs has also been associated with increased risk for binge drinking, tobacco use, intravenous drug use and risky sexual behaviors, such as not using a condom, in adulthood [12].

Increased risk for hazardous drinking among PWH has been documented, with two recent national studies of in-care PWH estimating that 32% (total sample N = 7686) and 26% (total sample N = 9336) screened positive for hazardous alcohol use as assessed by the Alcohol Use Disorders Identification Test (AUDIT-C) [15, 16]. Alcohol use is linked to poorer mental and physical health outcomes and an increased risk for subsequent misuse [17–19]. Alcohol consumption is negatively associated with ART adherence, immune function, and reliably demonstrated to accelerate HIV disease progression and chronic comorbidity [20, 21, 22].

When considering the mechanisms that link childhood adversity, hazardous drinking patterns, and health in PWH, it is important to recognize that alcohol use frequently changes across the life course [23]. ACE survivors are shown to initiate alcohol use at an earlier age [24, 25], which can lead to poor health outcomes and health-threatening behaviors [26]. Neurobiological studies have found that exposure to early life trauma and chronic stress alters biological pathways, such as the hypothalamo-pituitary (HPA) axis, glucocorticoid production, and dopaminergic (DA) response, resulting in enhanced dopamine release in response to psychostimulant administration, thus making trauma survivors more vulnerable to addiction [27]. Childhood trauma has not only been related to alcohol initiation but lifetime hazardous drinking and susceptibility to relapse [28]. Research from the *New Orleans Alcohol use in HIV* (NOAH) Study has found that a steeper increase in alcohol consumption after ages 10–20 was related to worse health-related quality of life, frailty, and current drinking [29]. Other studies using NOAH data have found that higher alcohol use at the time of data collection was related to liver disease, fibrosis, higher caloric and fat intake, and insulin resistance, while lifetime alcohol consumption was related to cardiometabolic risk factors and frailty risk [30–32].

Alcohol misuse, particularly as a maladaptive coping technique, is a modifiable health behavior with interventions demonstrating improvements in anxiety, depression, and social functioning [18, 33–35]. However, the evidence for effectiveness of interventions on HIV related outcomes (i.e., CD4+, viral load) is inconclusive. [36, 37]). Early-life trauma and ACEs, which are often predicated on childhood poverty [38], are, definitionally, non-modifiable in adulthood. Advancing robust models of trauma-informed, PWH-tailored, recovery from alcohol misuse will require sophisticated understandings of the inherent biopsychosocial dynamics that link ACEs with poorer health in later adulthood. Life course theory (LCT) [39]; alongside broader developmental systems frameworks, emphasizes the socio-environmental circumstances that may underlie age of alcohol initiation and onset of specific problem-drinking (e.g., bingeing) patterns [40, 41], particularly in the context of childhood economic hardship [24, 25, 38, 40]. As such, the aims of the present study were twofold: (1) to examine the associations between childhood adversity (ACEs and CEH), retrospectively captured lifetime drinking patterns and current health among adult in care PWH; and (2) to explore if these identified lifetime drinking patterns might serve as pathways from ACEs to poorer mental and physical health outcomes in PWH.

## Methods

### Data

These data were collected as part of the NOAH Study*,* a translational longitudinal study examining the interaction of alcohol use with biological and socio-environmental mechanisms impacting HIV-associated comorbidities, accelerated aging, and disease progression [42]. Adult PWH were recruited from an HIV outpatient clinic and a local federally qualified health center from October 2015 to October 2017. Potential participants were screened using the AUDIT and invited to enroll in the translational longitudinal study. To enroll a sufficiently powered sample to examine the impact of alcohol use on HIV progression comprehensively, respondents with AUDIT scores > 8 were oversampled. We provide a brief description of study recruitment and data collection here; greater detail is provided in a previous publication [42].

Study eligibility included non-pregnant PWH age 18 and older who were without acute illness, including non-prophylaxis prescription of antibiotics, or alcohol intoxication at the study visit. There were no further exclusion criteria. Once informed consent was obtained, participants attended a baseline visit at which data on residential address, alcohol use, physical and mental health, and other health-related factors were collected. Participants also provided blood, stool, vaginal, and saliva samples for analysis of HIV clinical outcomes and health biomarkers, blood pressure, and anthropometric measures. Details on the study protocol, recruitment strategy and measures have been published previously [42]. We used data from baseline interviews as well as medical chart abstraction in the present analysis (N = 365). The parent study was approved by the Louisiana State University Health Science Center (LSUHSC) and Tulane University Institutional Review Boards. All participants provided informed consent.

### Measures

*Childhood adversity* Two measures of childhood social and economic adversity were included in the present study. *Adverse childhood experiences* (*ACE*) were measured with the adverse childhood experiences questionnaire from the Kaiser Permanente ACE study [13]. In this questionnaire, persons are asked whether they experienced psychological, physical, or sexual abuse; witnessed violence against their mother/caregiver; or lived with household members who evidenced hazardous patterns of substance use, had a mental illness or were suicidal or were ever imprisoned during their childhood (before age 18). Total ACE score was calculated as the sum of all positive responses (range 0–10), with higher scores representing greater adverse experiences.

*Childhood economic hardship* was assessed with questions adapted from the U.S. Census Bureau’s Survey of Income and Program Participation (SIPP) [43]. Participants were asked if before age 12, their household was unable to pay the rent/mortgage; experienced eviction; was unable to pay gas, oil, or electricity bills; went hungry because there was not enough money for food; moved in with other people because of financial problems; and whether their family owned their own home (reverse coded). Responses were summed to yield a total score (range 0–6), with higher scores representing greater economic hardship.

*Life course alcohol use patterns* Patterns of alcohol use across the life course were captured via age at first alcohol use and lifetime drinking trajectories. *Alcohol use initiation age* was captured with a single question at baseline asking participants at what age they first drank alcohol. *Lifetime drinking trajectories* were constructed based responses to a modified version of the Lifetime Drinking History questionnaire [44]. This series of questions asks about average quantity (e.g., “When you were 10–20 years old, how much did you drink on a typical day when you were drinking?”) and frequency (e.g., “When you were 10–20 years old, how often did you drink?”) of alcohol consumption during each decade of life, starting with the onset of regular drinking and ending with the individual’s current drinking pattern. Its validity and reliability have been established in several previous studies with heavy and non-heavy drinkers [44, 45–47]. For each decade of life, quantity and frequency measures were multiplied to yield average annual number of drinks. These counts were divided by 12 to derive average monthly drinks during each decade, then log-transformed for trajectory analyses due to the right-skewness of the counts. Additional details about how we constructed lifetime drinking trajectories are provided in the “Analysis” section below.

*Health status* To capture general physical decline, three measures of physical health status that span physiological systems were included. These measures also demonstrated associations with lifetime alcohol use in prior studies with this cohort [29]. *Health-related Quality of Life* (*HRQoL*) was measured via responses to the Short Form 36 (SF36) [48]. This 36-item questionnaire measures eight health-related domains. In the present study, general perception of health (five items) was included. The five items were summed then transformed to a 0–100 scale (worst to best possible health state). *Frailty* was captured by a deficit index constructed from 58 items (DI58). Drawn from a prior study examining cumulative lifetime alcohol volume and frailty among NOAH participants, the deficit index included items that were based on self-report (e.g., “how would you rate your health”), screening tests (e.g., Mini-Mental State Examination), clinical evaluation (e.g., congestive heart failure), as well as laboratory measurement (e.g., diabetes mellitus) [49, 50]. Binary deficits were scored as either 0 or 1, whereas deficits with multiple response levels were evenly graded between 0 and 1. The sum of the health indicators was then divided by the total possible score of 58. Higher scores (possible range 0–1) indicate greater frailty. Lastly, a *multi-morbidity index* was included to provide an additional measure of co-morbidity in the sample. Multimorbidity is a geriatric syndrome defined as the co-prevalence of aging-associated medical conditions [51]. The NOAH Study Multimorbidity Index (MMI) is the sum of 14 common geriatric comorbidities (heart disease, cancer [excluding HIV malignancies], chronic lower respiratory diseases, stroke (cerebrovascular diseases), diabetes, chronic kidney disease, hypertension, hyperlipidemia, arthritis (any type), affective disorder, dementia, anemia (any etiology), osteoporosis, chronic liver disease) diagnosed in an individual participant.

Similarly, three measures of mental health status were also included. *Anxiety and depression* were measured using the Hospital Anxiety and Depression Scale (HADS), a validated and reliable screening tool consisting of 14 items (including 7-item subscales for anxiety and depression) [52, 53]. Though the continuous summary score was used in this analysis, a cut off of eight is considered ‘borderline’ while a cut off of 11 is considered ‘clinical’ for both anxiety and depression subscales [53]. PTSD symptoms were assessed using the Primary Care PTSD Screen (PC-PTSD-5) [54]. This instrument is a revision of the original PC-PTSD reflecting updates to PTSD diagnostic criteria in the *DSM-5*. The screener includes five yes/no items, summed for a possible score of 0–5. Though the continuous summary score was used in this analysis, a cut off of three is considered ‘borderline’ while four is considered ‘clinical’ [54]. This updated measure has demonstrated excellent diagnostic accuracy [54].

*Demographic controls* Several demographic control variables were explored, based on their correlation with childhood adversity, lifetime drinking trajectories, and/or health status. We focused on those which would be exogenous to and temporally precede childhood adversities, to avoid controlling for potential mediators. *Age* was included as a continuous variable, centered at the sample mean. Our past work demonstrated generational differences in lifetime drinking patterns, and health is generally known to decline with age [29]. Sex assigned at birth was included due to known sex differences in alcohol use patterns and health status [55, 56]. *Educational attainment* and *income* were also explored as potential confounders. However, these were eventually excluded both due to their non-significant relationships with study variables and due to concerns, that they could be products of childhood adversity and/or lifetime alcohol use patterns.

### Analysis

*Overall approach* We began analyses by generating descriptive statistics for all study variables. We then proceeded to construct the latent variables we would use for structural equation models. Lastly, we estimated the full structural equation model, inclusive of our primary predictors (ACE, childhood economic hardship); lifetime drinking mediators (age at first drink; lifetime drinking trajectory parameters); and our health status outcomes (physical health; mental health). Both direct and indirect paths between the predictors and outcomes were tested.

*Latent lifetime alcohol use trajectory parameters* Like past analyses [29], we characterized lifetime drinking trajectories applying latent curve modeling in MPlus [57]. Latent curve models are an extension of structural equation models which estimate growth parameters (intercept, slope, quadratic) purged of measurement error [58, 59]. The logged average monthly alcoholic drinks for each decade of life were input as observed indicators of the latent trajectory parameters. Full information maximum likelihood (FIML) estimation was used. The best fitting model was the quadratic model, which included intercept, linear slope, and quadratic (deceleration) parameters. For lifetime drinking history, the intercept represented the average number of drinks in the first decade measured (ages 10–20), the linear term represented the change (slope) in average number of drinks across decades over time, and the quadratic represented the change in slope of average number of drinks over time. However, due to instability of the variance around the quadratic parameter, its variance was set to zero. The intercept and slope were allowed to covary.

*Latent mental and physical outcomes* Physical and mental health were specified in our model as a pair of correlated latent variables. Indicators for each (physical health: HRQoL, frailty, and multimorbidity; mental health: anxiety, depression, and PTSD symptoms) were standardized to facilitate estimation, given widely different measurement scales. The latent health variables were scaled to have a mean of zero and a variance of one and were allowed to covary.

*Structural equation modeling* After establishing the fit of the latent components of the model, we proceeded to combine the elements into a comprehensive structural equation model, estimated using robust maximum likelihood. Demographic controls were entered as predictors of childhood hardship variables; lifetime drinking variables; and health status variables. Where associations between demographic variables and other variables were non-significant, demographic controls were trimmed for model parsimony. Final model fit was assessed via various fit indicators used in evaluation of structural equation models (i.e., RMSEA; CFI/TLI; etc.) [60].

## Results

*Descriptive statistics* Descriptive statistics are displayed in Table 1. Participants’ ages ranged from 20 to 71 with a mean of 48.2 (SD = 10.35). Most of the sample identified as Black (83.6%), male (69.0%), had a household income below $20,000 a year (88.74%) and over a third identified as a sexual minority (38.4%). In terms of education, 40.8% had less than a high school education, 31.2% had a high school diploma or GED, 22.2% had some college education, 3.8% had a 4-year college degree, and 1.9% had a graduate or professional degree. Predictors of childhood adversity included childhood economic hardship, which had a mean score of 1.5 (SD = 1.5) on a range of 0–6, and ACE score, which had a mean score of 3.4 (SD = 2.7) on a range of 0–10. The sample population had a mean number of 547.9 (SD = 989.9) drinks per year between ages 10 and 20, which increased to 790.3 (SD = 1167.2) drinks per year between ages 21 and 30. From ages 31 to 40, the mean number of drinks per year was 799.6 (SD = 1182.1), which decreased to 703.0 (SD = 1108.5) from ages 41 to 50, to 554.6 (SD = 936.5) from ages 51 to 60 and finally to 274.2 (SD = 570.0) from ages 61 to 70. A high proportion of respondents had borderline or clinical depression (27.7%), anxiety (42.2%) and/or PTSD (21.15%) compared to prevalence in the general population. For the physical indicators, participants reported an average of 3.12 multi-morbidities (SD = 1.77), had an average frailty score of 0.17 (SD = 0.087) out of 1 and a general perception of health score of 59.4 (SD = 23.3) on a scale of 0 (worst possible health) to 100 (best possible health).

**Table 1:** The New Orleans alcohol use in HIV (NOAH) sample: demographics, childhood adversity, and current health (N = 365)

		N (%)
Demographics and background factors		
Age (range 20–71)	Mean (SD)	48.2 (10.35)
Race (Black)		305 (83.6)
Sex (male)		252 (69.0)
Sexual minority		140 (38.4)
Education		
Less than high school		149 (40.8)
High school grad/GED		114 (31.2)
Some college		81 (22.2)
4-year college grad		14 (3.8)
Grad/professional school		7 (1.9)
Income < 20,000		323 (88.74)
Ever experienced homelessness		192 (52.60)
Ever been incarcerated		234 (64.11)

		Mean (SD)
Predictors (childhood adversity)		
Childhood economic hardship (range 0–6)		1.5 (1.5)
ACE score (range 0–10)		3.4 (2.7)
Mediators		
Age at first drink		16.99 (4.97)
Lifetime drinking history (average drinks/year, by decade)		
Ages 10–20 (range 0–3650)		547.9 (989.9)
Ages 21–30 (range 0–3650)		790.3 (1167.2)
Ages 31–40 (range 0–3650)		799.6 (1182.1)
Ages 41–50 (range 0–3650)		703.0 (1108.5)
Ages 51–60 (range 0–3650)		554.6 (936.5)
Ages 61–70 (range 0–2920)		274.2 (570.0)

		N (%)
Mental health indicators		
Borderline/high depression score		101 (27.7)
Borderline/high anxiety score		154 (42.2)
Borderline/high PTSD		77 (21.15)

		Mean (SD)
Physical health indicators		
SF 36 indictors		
General perception of health (range 0–100)		59.4 (23.3)
Multimorbidity Index		3.12 (1.77)
Frailty		0.17 (0.087)

*Structural equation modeling results* Fit statistics indicated that the model fit the data relatively well (CFI = 0.949, RMSEA = 0.043).

Table 2 details the indirect and total pathways from childhood hardship variables (CEH and ACES) to physical and mental health. Indirect pathways were significant for both economic hardship (β = 0.033; p = 0.029) and ACES (β = 0.009; p = 0.029) for mental health, but not for physical health. Total effects were significant for all four pathways with the largest effect size for ACEs to mental health (β = 0.147; p < 0.001).

**Table 2:** Indirect and total pathways from childhood adversity to health outcomes

Paths	β	S.E.	Est./S.E.	Two-tailed p value
Indirect effect on physical health				
ACE → AFD → LDH → PH	0.004	0.003	1.432	0.152
CEH → LDH → PH	0.014	0.01	1.453	0.146
Indirect effect on mental health				
ACE → AFD → LDH → MH	0.009	0.004	2.163	0.031
CEH → LDH → MH	0.033	0.015	2.189	0.029
Total effects				
Total effect of CEH on physical health	0.118	0.042	2.806	0.005
Total effect of ACEs on physical health	0.103	0.031	3.352	0.001
Total effect of CEH on mental health	0.126	0.051	2.476	0.013
Total effect of ACEs on mental health	0.147	0.033	4.527	< 0.000

*ACE* adverse childhood experiences, *CEH* childhood economic hardship, *AFD* age at first drink, *LDH* lifetime drinking history intercept, *PH* physical health, *MH* mental health

**Fig. 1:**
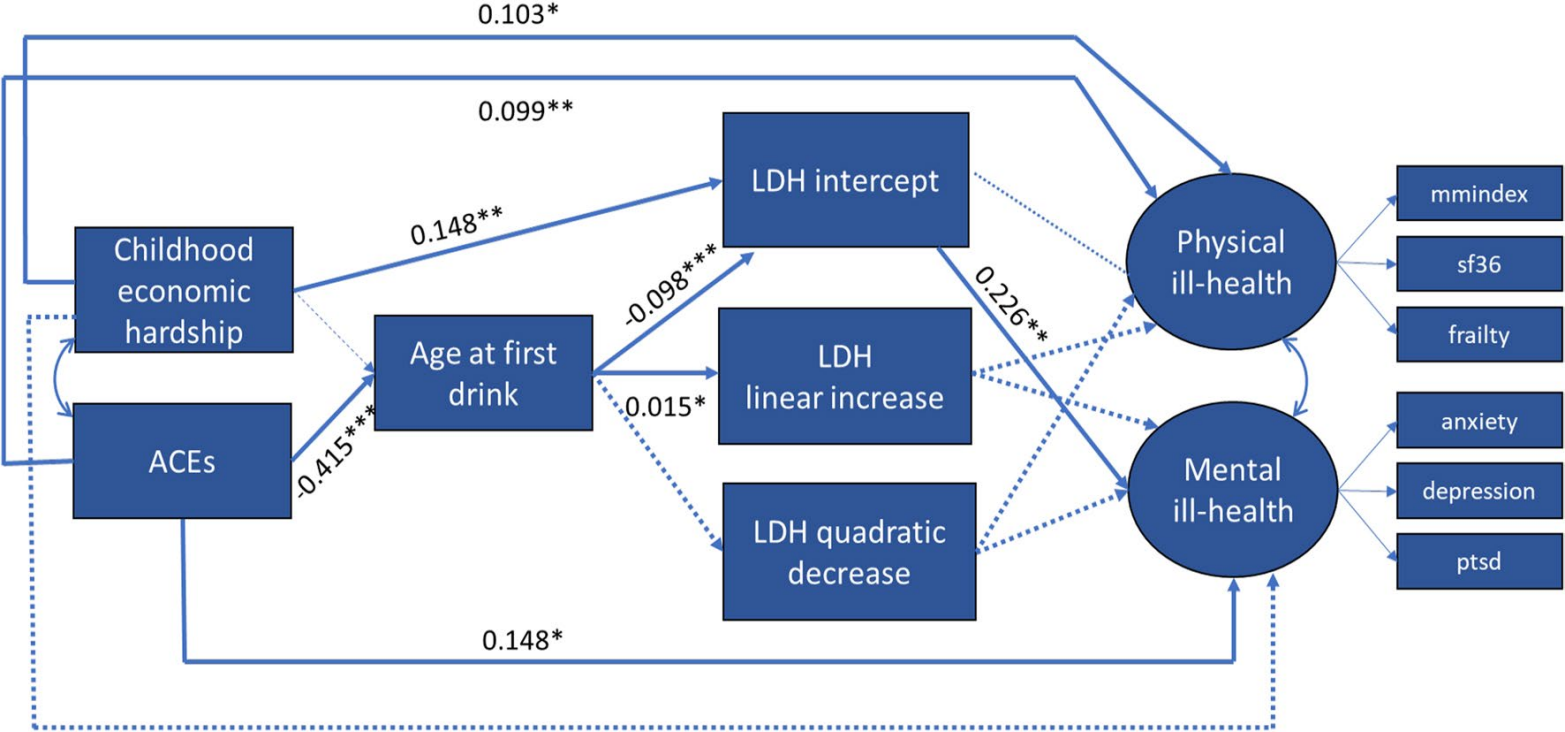
Structural equation mediation model. *p < 0.05; **p < 0.01;***p < 0.001; *ACE* adverse childhood experiences; *CEH* childhood economic hardship; *LDH* lifetime drinking history intercept. Purple arrows represent pathways from CEH while green arrows represent pathways from ACEs. Alternating green and purple lines represented shared pathways between ACEs and CEH. Dark solid lines represent significant relationships and lighter dotted lines represent tested for but insignificant relationships (Color figure online)

Results of the path analysis are presented in Fig. 1. In this model ACEs were significantly and negatively associated with age at first drink (those with higher ACE scores initiated drinking sooner) (β = − 4.15; p < 0.001). Childhood economic hardship was associated with lifetime drinking history intercept (those with higher childhood economic hardship scores were more likely to engage in heavier drinking between ages 10 and 20) (β = 0.148; p = 0.001). Age at first drink was in turn associated with the lifetime drinking history intercept (β = − 0.098; p < 0.001) and lifetime drinking history linear increase (β = 0.015; p = 0.037). Lifetime drinking history intercept was in turn associated with mental health (β = 0.226; p = 0.003).

## Discussion

This study explored pathways between childhood adversities (ACEs and CEH), lifetime alcohol use, and mental and physical health outcomes among PWH enrolled in the NOAH study, embracing a lifecourse-informed approach. Our findings revealed that ACES and CEH have a significant impact on both lifetime drinking patterns and current health, particularly mental health, in our study sample. Drinking history early in life (ages 10–20) was a significant mediating factor for the relationship between childhood adversity and mental health with the strongest mediation effects for CEH, indicating the strength of the relationship between childhood poverty, patterns in alcohol use, and mental wellbeing.

These findings build on prior work exploring the detrimental impacts of familial poverty and ACEs on health-related challenges in later adulthood [1–14, 24–26]. Prior research has outlined negative health impacts of various forms of ACES and CEH in both domains of mental health (PTSD, depression, etc.) and physical health (cardiovascular health, nutrition, etc.) [2, 6–14]. This body of research emphasizes the influence of ACES and CEH on brain development and cognitive processes, highlighting the importance of mental health both as a factor underpinning pathways between adversity and physical health and as an avenue for intervention [3, 6, 9].

We found significant associations between ACEs, CEH and early life alcohol use, with early alcohol use mediating ACEs and CEH’s impact on later life health. Some studies have found connections between ACEs, socioeconomic status, and substance use initiation in adolescent populations [24, 61, 62]. However fewer studies examine drinking frequency trajectories across the life course. Prior work using data from the NOAH study has found that a larger increase in alcohol consumption following ages 10–20 was associated with worse health related quality of life and that high levels of alcohol consumption across the life course was related to cardiometabolic factors and liver functioning [29, 30]. Multiple studies suggest that all but the most severe physical effects of heavy alcohol use in adults including fatty liver, GI tract dysregulation, and myocardial damage are reversible with 4–6 weeks of abstinence from alcohol [63–65].

Additionally, though we found mediating effects of drinking trajectories on the pathways between ACES and CEH and health, these mediating effects were strongest for CEH. Recent meta-analytic findings demonstrate consistent, divergent, processes of premature biological aging associated with “deprivation” versus “threat” exposures in childhood, indicating potentially different pathways for CEH (‘deprivation’) and childhood maltreatment (‘threat’) on health [6, 66]. Alcohol use, particularly among PWH reaching older adulthood, may accelerate these processes [67].

More research is needed to explore the long-term impacts of heavy alcohol use on adolescents; however, studies have indicated long-lasting neurodevelopmental changes including elevated neuroinflammation and altered hippocampal neurogenesis, which are related to learning, memory, social and mood functioning, even after reduced use in adulthood [68–70]. This may explain why mediating pathways of early life alcohol use were more strongly associated with later life mental health, indicating that recovery for mental health concerns may necessitate further intervention beyond abstinence.

### Practice Implications

Our findings suggest that accumulating ACEs during childhood is associated with earlier alcohol initiation, average number of drinks consumed in adolescence, and particularly strong negative impacts to mental health (in addition to their impacts on physical health). As early life history variables were tied to later life health, these findings support lifecourse-informed approach to health and particularly mental health in this population. Through this lens, intervening on alcohol mis/use in the U.S. must consider that alcohol is likely adopted as a maladaptive coping technique in response to early life economic hardship and social-emotional stressors. This chronic stress over the life course can undermine the development of long-term healthy stress responses [41, 71, 72].

Treatment strategies for poor mental health and maladaptive coping frequently focus on adaptive coping skills and behavior change. However, interventions informed by these findings must also acknowledge the broader systemic/structural factors and personal histories which shape the lives of PWH [73]. One example of such an intervention; the Intervention Innovations Team, integrated conceptual model (IIT-ICM), developed by Gwadz et al., incorporated critical race theory, harm reduction, and self-determination theory along with more traditional behavior change intervention components to better serve the complex needs of Black and Latino PWH enrolled in the Heart to Heart 2 project [73]. This approach, which emphasized person-centeredness with an understanding of upstream historical, structural, and systemic factors which occur across the life course was identified to be highly acceptable and lacking in most HIV care settings by Heart to Heart 2 participants [73]. Further, screening for ACEs and trauma history has received increasing attention in the HIV care field due to demonstrated effectiveness in increasing linkage to trauma-informed and wraparound care [74]. In addition to incorporating trauma histories in HIV care, a truly lifecourse-informed approach recognizes the importance of comprehensive multisectoral service systems: (1) “vertically,” or across primary, secondary, and tertiary care; (2) “horizontally” in wraparound coordination with social services such as housing (a common and overwhelming stressor in many in-care PWH [75, 76]); and, (3) “longitudinally,” building continuity of pediatric, adolescent, transition-age, and adult healthcare [77]. Continuity of high-fidelity wraparound pediatric, adolescent, and early adulthood healthcare carries the potential to disrupt multiple harmful health behaviors in youth, including those associated with HIV acquisition [78, 79], early alcohol initiation, and hazardous alcohol-use behaviors within the critical 10–20-year-old developmental span implicated by our findings. Additionally, strengthening the family economically through job training, housing vouchers and assistance may dismantle “toxic” stressors that arise from CEH [71, 80–82].

### Policy Implications

Though a lifecourse-informed approach encourages early intervention on childhood trauma and economic hardship on a systemic level, interventions remain incumbered by fiscal, bureaucratic [83, 84], and political obstacles [85], particularly across the Gulf South. Louisiana, for example, is mired in family economic hardship: statewide, 18.0% of households earned incomes below the federal poverty line (FPL), and an additional 33.0% could not afford basic costs of living [86, 87] with ~ 140,000 children enduring “deep poverty” (< 50% FPL [88]) prior to the COVID-19 pandemic [89, 90]. Political economy scholars highlight Louisiana policymakers’ refusal to raise the state minimum wage, intersecting with longstanding nationwide lack of paid parental leave, subsidized childcare, and affordable housing [91, 92], as contributing to growing inequality in the state [81, 82]. Viewed through a lifecourse lens, persistent poverty in Louisiana, the structural context driving ACE accumulation, and the lifelong health challenges for PWH result from intentional policymaking [1–10, 24, 25] that even the most carefully tailored models of individual treatment may only partially ameliorate.

### Limitations

This study has several strengths, including a lifecourse-informed approach and the combination of latent curve and structural equation modeling, which allowed us to analyze complex relationships to capture both within-individual variability (e.g., change over time in alcohol use) and between-individual variability (e.g., differences in initial levels of alcohol use) in a path analysis. However, there are several limitations to note. First, measurements were taken using a single survey and ACEs, lifetime economic hardship, and alcohol patterns were captured retrospectively. Concerns about recall bias arise from retrospective surveys around alcohol use, particularly as some research suggests this design underestimates alcohol consumption by at least 30% [93]. However other studies have found that retrospective lifetime drinking history assessments are highly to moderately correlated with prospective measures and with health outcomes in the anticipated direction [44–47, 94]. Similar concerns regarding retrospective childhood maltreatment measures state that mental health conditions such as depression interfere with recall. However, a recent study found changes in repeat measures of ACE scores to be unrelated to mental health [95]. Secondly, it was necessary to set the variance of the quadratic term to zero due to low variance in the sample, meaning that the quadratic term was held constant over time for everyone. Though fixing the quadratic term to zero can simplify the model, it may also lead to risks for misspecification [96]. Third, physical and mental health were assigned as correlated latent variables. While the measures we chose are commonly studied for both mental and physical health, they are not comprehensive of an individual’s overall physical and mental wellbeing. Lastly, respondents were recruited from HIV clinics, as such the sample represented only in care PWH. This is significant as substance use is known to impact engagement across the continuum of HIV care, therefore our sample population may not fully or accurately represent HIV positive substance users [97].

## Conclusion

Our study, providing a LCT informed perspective, adds to the current body of literature on childhood adversity, lifetime alcohol use, and later life mental and physical health outcomes. Respondents in our sample not only reported high levels of ACEs, CEH, depression, anxiety, and PTSD, but also experienced homelessness (52%) and incarceration (64.11%) during their lifetime at high rates, and the majority (88.74%) had household incomes below 20,000 USD a year. This, along with our findings that CEH and ACEs were related to adolescent and young adult alcohol use patterns, which were in turn associated with later life health, suggests a need for interventions which include recognition of the pervasive toll of poverty and trauma in the lives of in-care PWH who use or misuse alcohol [10–38, 40]. Along with behavior change interventions, this includes high fidelity wraparound healthcare across the life course [76] and preventative interventions targeted at the emotional and financial well-being of children and families [70, 71, 78, 79]. Ultimately an environment of high poverty and inequity, as is the current climate in Louisiana, undermines attempts by integrated healthcare systems to address trauma in a meaningful way. As such, addressing poverty and systemic issues at the policy level is necessary to address childhood trauma and improve health outcomes for PWH in Louisiana.

## Data Availability

Not applicable.
